# Changes in Phospholipid Composition Studied by HPLC and Electric Properties of Liver Cell Membrane of Ethanol-Poisoned Rats

**DOI:** 10.1080/15376510701624035

**Published:** 2008-06-23

**Authors:** Barbara Szachowicz-Petelska, Izabela Dobrzyńska, Elżbieta Skrzydlewska, Zbigniew A. Figaszewski

**Affiliations:** Institute of Chemistry, University in Białystok, Al. Piłsudskiego 11/4,15-443, Białystok, Poland; Department of Analytical Chemistry, Medical Academy of Białystok, 15-230, Białystok 8, Poland; Institute of Chemistry, University in Białystok, Al. Piłsudskiego 11/4,15-443, Białystok, Poland; and Laboratory of Interfacial Electrochemistry, Faculty of Chemistry, University of Warsaw, Pasteur St. 102-093, Warsaw, Poland

**Keywords:** Electric Charge, Ethanol, Green and Black Tea, Phospholipids

## Abstract

Ethanol introduced into the organism undergoes rapid metabolism to acetaldehyde and then to acetic acid. The process is accompanied by formation of reactive oxygen species (ROS), which damage mainly lipids of membrane cells. The effects of ROS can be neutralized by administering preparations with antioxidant properties. The natural preparations of this kind are teas.

This paper reports data on the effect of green and black tea on the surface charge density, content of phospholipids, and level of lipid peroxidation products of liver cell membrane of rats chronically intoxicated with ethanol. Surface charge density of liver cells was measured by the electrophoresis method, whereas qualitative phospholipid composition was determined by the HPLC method.

Ethanol administration caused an increase in the amount of all phospholipids, in surface charge density as well as in lipid peroxidation products. Ingestion of green and black tea with ethanol partially prevented these ethanol-induced changes, and the action of green tea was stronger than that of black tea.

## INTRODUCTION

About 90% of ethanol is oxidized in the liver, so acute and chronic ethanol intoxication is characterized by structural alterations of the liver cells. There, ethanol is oxidized into acetaldehyde and next into acetate; these processes are accompanied by free radical generation (Lieber 1997). Electrophilic free radicals and acetaldehyde readily react with the nucleophile groups of proteins, phospholipids, and nucleic acids to produce adducts, some of which have been detected in the tissues of alcoholic patients (Niemela et al. 1998; Nagata 1998). As a consequence of ethanol intoxication, conducive changes in the properties of the liver, including cell membrane properties, take place. The biological membrane functions as a selective barrier and is essential for transport, ion permeability, enzyme activity, and receptor responsiveness (Gennis 1989). All of these membrane functions require structural integrity and the accurate electric properties, which may be modified by changes in membrane composition. An important electric property of a biological membrane is its surface charge and its electrokinetic potential (i.e., the potential drop between the membrane and its environment) (Gennis 1989; Dołowy 1984; Tien 1974). Electric properties of the membrane depend on acid-base and complex formation equilibria between cell membrane components and the environmental components.

Because most of the changes caused by alcohol are directly or indirectly linked with lipid peroxidation, potent antioxidants are looked for, especially those found in natural products. One such potentially health-promoting beverage is tea, green as well as black. It is generally believed that teas have an influence on the effects of various biological and pharmaceutical processes, although many of them remain to be investigated. Green tea contains large amounts of polifenols, especially catechin, which possesses strong antioxidant properties (Skrzydlewska et al. 2002). However, main black tea components belong to oxidized polifenols named teaflavins and also have unexpected antioxidant abilities (Leung et al. 2001).

Therefore, the aim of this work is to compare the influence of green and black tea on membrane electric charge, phospholipid composition, and level of lipid peroxidation products of rat liver cells chronically intoxicated with ethanol.

## MATERIALS AND METHODS

### Green and Black Tea

Green tea, *Camellia sinensis (Linnaeus) O. Kuntze* (standard research blends–lyophilized extracts), was provided by TJ Lipton (Englewood Cliffs, NJ) and was dissolved in the drinking water at a concentration of 3 g/L. Fresh tea was prepared three times a week and stored at 4°C. The content of drinking vessels was renewed every day. Green tea extract contained epigallocatechin gallate (337 mg/L), epigallocatechin (268 mg/L), epicatechin (90 mg/L), and epicatechin gallate (60 mg/L), which were determined by the HPLC method (Maiani et al. 1997).

Black tea, *Camellia sinensis (Linnaeus) O. Kuntze* (standard research blends–lyophilized extracts), was provided by TJ Lipton (Englewood Cliffs, NJ) and was dissolved in the drinking water at a concentration of 3 g/L. Fresh tea was prepared three times a week and stored at 4°C. The content of drinking vessels was renewed every day. Black tea extract contained both the catechins–epigallocatechin gallate (EGCG), 14.53 mg/L; epigallocatechin (EGC), 2.21 mg/L, and epicatechin (EC), 2.83 mg/L–and the theaflavins (TFs)–theaflavin (TF_1_), theaflavin 3-gallate (TF_2_A), theaflavin 3′-gallate (TF_2_B), and theaflavin 3,3′-digallate (TF_3_)–in the amount of 156.16 mg/g dried extract. The levels of catechins and theaflavins were determined by modified HPLC methods of Mattila et al. (2000) and Lee et al. (2000).

### Animals

Twelve-month-old male Wistar rats used for the experiment had free access to a granular standard diet and water and were maintained under a normal light-dark cycle.

The animals from the green tea part of the experiment were divided into the following groups:

**Control group 1** was treated intragastrically with 1.8 mL of physiological saline every day from 6 to 10 weeks.
**Green tea group** received Lipton green tea solution (3 g/L) ad libitum instead of water from 5 to 10 weeks.
**Ethanol group 1** was treated intragastrically with 1.8 mL of ethanol in doses from 2.0 to 6.0 g/kg b.w. every day from 6 to 10 weeks and the dose of ethanol was gradually increased by 0.5 g/kg b.w. every 3 days.
**Ethanol 1 and green tea group** received Lipton green tea solution (3 g/L) ad libitum instead of water from 5 to 10 weeks and was treated intragastrically with ethanol as the ethanol group.

The animals from the black tea part of the experiment were divided into the following groups:

**Control group 2** was treated intragastrically with 1.8 mL of physiological saline every day from 6 to 10 weeks.
**Black tea group** received Lipton black tea solution (3 g/L) ad libitum instead of water from 5 to 10 weeks.
**Ethanol group 2** was treated as ethanol group 1.
**Ethanol 2 and black tea group** received Lipton black tea solution (3 g/L) ad libitum instead of water from 5 to 10 weeks and was treated intragastrically with ethanol as ethanol group 2.

All procedures were in accordance with Guide for Care and Use of Laboratory Animals and the local Animal Care Committee approved the protocol.

### Electrochemical Methods

In order to determine surface charge density of cell membrane, liver cells were put into the measuring vessel and electrophoretic mobility was measured by using the DTS5300 ZETASIZER 3000 apparatus (Malvern Instruments).

The surface charge density has been determined using the equation: σ = ηu/d, where u is the electrophoretic mobility, η the viscosity of solution, and d the diffuse layer thickness (Krysinski and Tien 1986). The diffuse layer thickness was determined from the formula (Sobczyk et al. 1982) $d=\sqrt{\frac{\varepsilon \cdot \varepsilon_{0}\cdot R\cdot T}{2\cdot F^{2}\cdot I}}$, where R is the gas constant, T is the temperature, F is the Faraday number, I is the ionic strength of 0,9% NaCl, and ɛɛ_o_ is the permeability electric medium.

### Isolation and Analysis of Phospholipids by HPLC Method

The tissues (about 1.5 g) were homogenized in 1 mM-NaHCO_3_ (pH = 7.6), 0.5 M CaCl_2_ in a loose-fitting Dounce homogenizer. Membrane fragments were separated from nuclei and mitochondria by rate-zonal centrifugation of the “low-speed” pellet as described by Evans (1970). The sediment was washed and partially separated in two consecutive centrifugations at 1000 × g. The sediment was homogenated in saccharose of 1.22 density and in the next step was covered with saccharose of 1.16 density. The cell membranes were separated by centrifugation at 2000 × g for 25 to 35 min. Next, phospholipids were extracted by a Folch method. Tissue was homogenized in a chloroform–methanol mixture of 2:1 volume ratio using 20 cm^3^/g of tissue. The solution was next filtered out with degreased paper filters and the precipitate was washed with the extracting solution containing 0.05 M calcium chloride; the volume ratio of chloroform, methanol, and aqueous calcium chloride solution was 8:4:3. The suspension was centrifuged at 500 × g for 2 min; the organic and the aqueous phases were separated; the aqueous phase was shaken again with chloroform, methanol, and water mixture of 3:48:47 volume ratio; and the phases were separated. The organic phases were combined and dried by evaporation to dryness. The extract was dissolved in 200 μL of chloroform–methanol mixture (3:2) (Szachowicz-Petelska et al. 2002).

The HPLC analysis was next carried out. The isolated phospholipids were separated by group analysis using NP-HPLC (liquid chromatography in normal phase system) in silica gel column. An acetonitryle–methanol–85% phosphoric acid mixture of 130:5:1.5 volume ratio was used as the eluent in an isocratic elution at 1 mL/sec flow rate and at 214 nm wavelength (Ostrowska et al. 2000).

### Biochemical Analysis

The extent of lipid peroxidation in liver cells was assayed with thiobarbituric acid (TBA). Chromogenous condensation product of TBA with malondialdehyde (thiobarbituric acid-reactive substances [TBARS]) was extracted from the aqueous phase into butanol and then an absorption at 532 nm was monitored (Buege and Aust 1978). The concentration of malondialdehyde was expressed in nmol TBARS/mL liver homogenates.

### Statistical Analysis

The data obtained in this study are expressed as mean ± SD. The data were analyzed by use of standard statistical analyses: one-way ANOVA with Scheffe's F-test for multiple comparisons to determine any significance between different groups. The values for *p* <0.05 were considered significant.

## RESULTS

Table 1 shows changes in the content of phospholipids and their surface concentration in liver cell membrane from control rats and animals that received ethanol or ethanol and green tea (or black tea). Both green and black tea ingestion did not significantly affect the content of phosphatidylinositol (PI), phosphatidylserine (PS), phosphatidylethanolamine (PE), and phosphatidylcholine (PC) and their surface concentration. Ethanol intoxication caused an increase in the PI, PS, PE, and PC content in the liver cell membrane and in their surface concentration. The above parameters characterizing PI, PS, PE, and PC increased by about 190%, 250%, 200%, and 200%, respectively, for the rats from the green tea part of the experiment and by about 170%, 260%, 150%, and 200%, respectively, for the rats from thee black tea part of the experiment. Administration of alcohol to green tea (or black tea) rats causes a smaller increase in the cited parameters characterizing PI, PS, PE, and PC than administration of ethanol only. The increase was smaller by about 30%, 10%, 60%, and 70% in the amount of these phospholipids for the rats that were administered ethanol and black tea than for the rats that were administered ethanol and green tea, where it was by about 100%, 80%, 80%, and 100%, respectively, when compared with the ethanol groups.

**TABLE 1:** Effect of green and black tea on content of phospholipids and of their surface concentrations in liver cell membrane of rats intoxicated with ethanol

Groups	Phospholipids	Content of phospholipids of plasmalemma (mg/g tissues)	Surface concentration of phospholipids (10^−7^ mol/m^2^)	Groups	Content of phospholipids of plasmalemma (mg/g tissues)	Surface concentration of phospholipids (10^−7^ mol/m^2^)
Control 1	PI	0.023 ± 0.003	0.10 ± 0.01	Control 2	0.022 ± 0.002	0.09 ± 0.02
	PS	0.026 ± 0.003	0.12 ± 0.03		0.025 ± 0.004	0.12 ± 0.01
	PE	0.392 ± 0.023	1.74 ± 0.09		0.367 ± 0.020	1.63 ± 0.07
	PC	0.426 ± 0.031	2.00 ± 0.01		0.419 ± 0.010	1.96 ± 0.06
Green tea	PI	0.024 ± 0.008^b^	0.10 ± 0.02^b^	Black tea	0.022 ± 0.007^y^	0.09 ± 0.04^y^
	PS	0.027 ± 0.003^b^	0.13 ± 0.01^b^		0.026 ± 0.001^y^	0.12 ± 0.05
	PE	0.401 ± 0.011^b^	1.78 ± 0.07^b^		0.397 ± 00.010^y^	1.76 ± 0.09^y^
	PC	0.432 ± 0.079^b^	2.02 ± 0.09^b^		0.427 ± 0.080^y^	2.00 ± 0.08^y^
Ethanol 1	PI	0.068 ± 0.010^a^,^c^	0.29 ± 0.02^a^,^c^	Ethanol 2	0.060 ± 0.009^x^,^z^	0.25 ± 0.06^x^,^z^
	PS	0.092 ± 0.019^a^,^c^	0.43 ± 0.05^a^,^c^		0.090 ± 0.013^x^,^z^	0.42 ± 0.07^x^,^z^
	PE	0.920 ± 0.108^a^,^c^	4.08 ± 0.97^a^,^c^		0.898 ± 0.121^x^,^z^	3.98 ± 0.10^x^,^z^
	PC	1.299 ± 0.113^a^,^c^	6.09 ± 1.07^a^,^c^		1.311 ± 0.110^x^,^z^	6.15 ± 0.44^x^,^z^
Ethanol + green tea	PI	0.032 ± 0.012^b^	0.14 ± 0.03^a^,^b^	Ethanol + black tea	0.047 ± 0.016^x^,^z^	0.20 ± 0.02^x^,^z^
	PS	0.050 ± 0.011^a^-^c^	0.61 ± 0.06^a^-^c^		0.078 ± 0.007^x^,^z^	0.36 ± 0.03^x^,^z^
	PE	0.490 ± 0.213^b^	2.18 ± 0.09^a^,^b^		0.560 ± 0.073^x^-^z^	2.49 ± 0.08^x^-^z^
	PC	0.632 ± 0.113^a^-^c^	2.96 ± 0.08^a^-^c^		0.768 ± 0.101^x^-^z^	3.61 ± 0.17^x^-^z^

PI, phosphatidylinositol; PS, phosphatidylserine; PE, phosphatidylethanolamine; PC, phosphatidylcholine. Statistically significant differences for *p* < 0.05.

a In comparison with control 1.

b In comparison with ethanol group 1.

c In comparison with green tea group.

x In comparison with control 2.

y In comparison with ethanol group 2.

z In comparison with black tea group.

**FIGURE 1:**
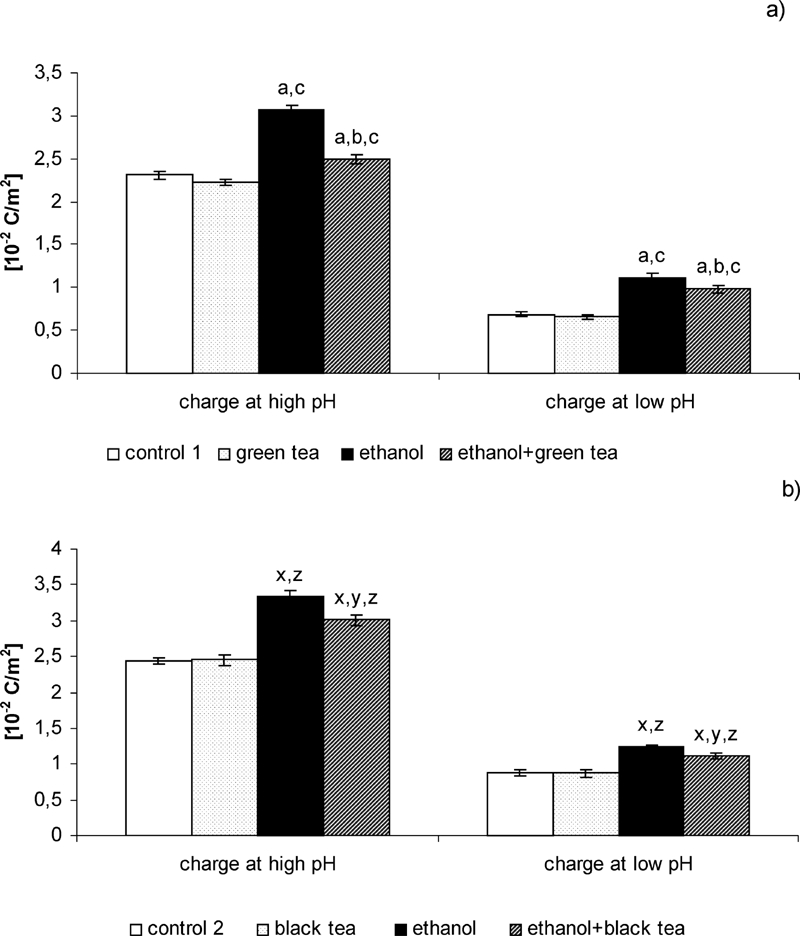
Effect of green (a) and black (b) tea on changes in electric properties of liver cell membrane in rats intoxicated with ethanol. (a): Statistically significant differences for *p* <0.05. ^a^In comparison with control 1. ^b^In comparison with ethanol group 1. ^c^In comparison with the green tea group. (b): Statistically significant differences for *p* <0.05. ^x^In comparison with control 2. ^y^In comparison with ethanol group 2. ^z^In comparison with the black tea group.

Figure 1 shows changes in surface charge density of liver cell membrane from control rats and animals that received ethanol or ethanol and green tea (or black tea). Ethanol intoxication of the rats caused an increase in negative charge by 35% at low pH values as well as in positive charge by 50% at high ones compared with the control rats. No significant differences were found in surface charge density of liver cell membrane of the rats watered with green tea or black tea compared with the control group. On the other hand, administering green tea or black tea with alcohol to the rats caused a considerable decrease in negative charge of liver cell membrane at high pH values (23% or 11%, respectively) and in positive charge at low ones (14% or 10%, respectively) compared with the animals to which alcohol alone was administered.

Treatment of rats with ethanol caused a 70% increase in the amount of lipid peroxidation products, measured as TBARS (Table 2). On the other hand, administering green tea or black tea with alcohol to rats caused a considerable decrease in the amount of lipid peroxidation products (29% or 21%, respectively) compared with the animals to which alcohol alone was administered.

**TABLE 2:** Effect of green and black tea on changes of malondialdehyde measured as thiobarbituric reactive substances (TBARS) of liver cells in rats intoxicated with ethanol

	Control 1	Green tea	Ethanol	Ethanol + green tea	Control 2	Black tea	Ethanol	Ethanol + black tea
TBARS (nmol/g tissue)	47.8 ± 2.5	39.5 ± 2.7^a^	81.5 ± 5.4^a^,^c^	63.1 ± 4.6^a^-^c^	45.4 ± 2.7	39.9 ± 2.4^x^	80.2 ± 6.2^x^,^z^	66.4 ± 5.1^x^-^z^

Statistically significant differences for *p* < 0.05.

a In comparison with control 1.

b In comparison with ethanol group 1.

c In comparison with green tea group.

x In comparison with control 2.

y In comparison with ethanol group 2.

z In comparison with black tea group.

## DISCUSSION

Phospholipids and proteins are the main charge carriers of biological membranes. The integral part of the membranes determining their structure is phospholipids. An effort has been made to evaluate the influence of individual phospholipids on the electric charge by chromatographic determination of their amounts. The results presented in this paper demonstrate that the changes in electric charge of the membrane are brought about variations in the amounts of phospholipids. The charge of phospholipids is mainly due to the amino groups in acidic medium (i.e., at low pH) and due to the carboxy groups and phosphate groups (i.e., at high pH). Results of this paper (Table 1 and Fig. 1) indicate that ethanol increases the content of individual phospholipids in the rat liver cell membrane. They are also supported by the literature data (Branchey and Buydens-Branchey 1990; Slater et al. 1993). It is known that ethanol influences the activity of enzymes participating in phospholipid (particularly of PE and PC) metabolism. Ethanol enhances the activity of acyltransferase that participates in phospholipid biosynthesis and decreases activity of phospholipase A_2_ that initiates phospholipid catabolism (Lindi 1998; Sun and Sun 1985). Moreover, ethanol may also increase the level of diacylglycerol, which takes part in phospholipid resynthesis (Podo 1999). In consequence, ethanol intoxication may be conducive to increases in phospholipid levels. Changes in membrane phospholipid composition are accompanied by the changes in surface charge and the discussed results indicate that an increase in the amounts of individual phospholipids provokes an increase in surface charge density of rat liver cell membrane (Table 1 and Fig. 1). It is connected with the increase in the amount of phospholipids that enhances generation of the new functional groups, both positively and negatively charged, to appear at the membrane surface. Ethanol causes a higher increase in phosphatidylserine and phosphatidylcholine content in liver cell membrane of rats than in other phospholipids. The amount of phosphatidylcholine is greatest in the outer liver cell membrane layer and this phenomenon can provoke a charge increase, both negative at high pH values and positive at low pH.

Ethanol and its oxidation products (acetaldehyde and acetate) influence the structure and physicochemical properties of rat liver cell membrane in different ways. Ethanol is likely to cause dehydration of the liver membrane cell surface. Acetaldehyde is a very reactive compound and does not occur in a free state in biological systems. It easily reacts with ɛ-amino groups of lysine, with SH groups of cysteine, and with methionine, arginine, and tyrosine residues of proteins. These reactions lower the positive charge of proteins, thereby increasing the negative charge of the membrane surface. Moreover, the conformation of membrane protein is maintained by a hydrophobic and hydrogen bond, and requires a hydrophobic lipid environment for the formation and stability of the secondary structure of hydrogen bonds (Klemm 1990; Klemm and Yuritas 1992). The perturbation of the protein structure may greatly affect lipid-protein interactions (Scott and Rabito 1988). Changes in membrane structure caused by the modification of lipid and protein structure lead to impairment in the structure of the hepatocyte membrane skeleton. One such known alteration is the change in distribution of phosphatidylserine, which is a component of the skeleton, from the internal to the external side of membrane (Tyurina et al. 2000). This can also cause an increase in the negative charge density of the membrane.

Ethanol intoxication is also accompanied by formation of free radicals that react with proteins as well as lipids, causing oxidative modifications of these membrane constituents. The enhanced level of lipid peroxidation products has been observed in this study after ethanol intoxication (Table 2). Lipid peroxidation products reduce hydrophobicity of the lipid cell membrane interior and modify the bilayer organization, thereby modifying physical properties of cell membranes and influencing membrane fluidity (Lindi et al. 1998; McGrath et al. 1995). Moreover, the ultimate consequence of oxidative modification of membrane proteins can be their aggregation or fragmentation (Dean 1995). Protein fragmentation creates new functional groups, both acidic and basic. The process can also expose membrane phospholipid functional groups hitherto screened by proteins. The changes can yield higher negative charge at high pH values and lower positive charge at low pH, as has been confirmed in this work.

Because most of the above changes appearing in liver membrane cells may be linked to the increased oxidative stress caused by an imbalance in the generation and neutralization of free radicals, these negative results may be compensated by the addition of exogenous substances possessing antioxidant properties, such as teas, both green and black (Luczaj et al. 2006; Ostrowska et al. 2005). The basic components of green tea, which have antioxidant properties, are catechins and their derivatives, whereas black tea contains mainly oxidized polyphenols–theaflavins and thearubugins (Rice-Evans et al. 1996; Luczaj and Skrzydlewska 2005). It has been recently revealed that teaflavins contained in black tea possess similar antioxidant properties as green tea catechins (Leung et al. 2001). Results from this paper indicate that in in vivo conditions the protective-antioxidative action of black tea toward liver cell membrane is only a little smaller than green tea.

Catechins as well as theaflavins possess the ability to prevent free radical generation, via inhibiting activity of enzymes participating in their generation, xanthine oxidase in particular, the enzyme that catalyzes oxidation of hipoxanthine and xanthine to uric acid accompanied by oxygen reduction to superoxide radical and hydrogen superoxide (Xianglin et al. 2000). Moreover, tea polyphenolic compounds can chelate transition metal ions like iron and copper to prevent their participation in Fenton and Haber-Weiss reactions, which in turn preclude the generation of hydroxyl radicals and degradation of lipid hydroperoxides causing reactive aldehyde formation (Frei and Higdon 2003; Middleton et al. 2000). Black tea has been demonstrated to inhibit iron absorption more effectively than green tea (Hollman and Katan 1997).

Protective action of teas is connected with the ability of polyphenols to scavenge free radicals including the most active hydroxyl radical, which may initiate lipid peroxidation. The oxidative attack from the aqueous phase seems to be an important reaction for initiating membrane lipid peroxidation and perhydroxyl radicals are regarded as the most feasible radicals for initiating lipid peroxidation in vivo.

Teas polyphenols can also reduce the mobility of free radicals in the lipid bilayer. Consequently, the decreased membrane fluidity results in inhibition of lipid peroxidation due to a slower pace of free radical reactions. They can penetrate the lipid bilayer influencing antioxidant capability in biomembranes. Catechins preferentially enter the hydrophobic core of the membrane where they exert a membrane-stabilizing effect by modifying the lipid packing order. They can also conserve the α-tocopherol content and delete the lipid peroxidation when membrane phospholipids are exposed to oxygen radicals from the aqueous phase. It has been shown that tea extracts as well as individual tea components, which also cross the blood-brain barrier, also protect erythrocytes and brain lipids phospholipids against oxidative modifications (Halder and Bhaduri 1998; Dobrzynska et al. 2005).

In conclusion, long-term drinking of green or black tea partially prevents changes in structure and function in liver cell membrane caused by chronic ethanol intoxication. Additionally, green tea supports the notion that its antioxidant properties contribute to a more important role in the health protection and disease prevention than black tea.
